# Vol.15 (2005), Supplement_II pp.S126

**DOI:** 10.2188/jea.15.197_4

**Published:** 2005-09-27

Wrong:BACKGROUD

Right:BACKGROUND

